# Cross- and Co-Packaging of Retroviral RNAs and Their Consequences

**DOI:** 10.3390/v8100276

**Published:** 2016-10-11

**Authors:** Lizna M. Ali, Tahir A. Rizvi, Farah Mustafa

**Affiliations:** 1 Department of Microbiology & Immunology, College of Medicine and Health Sciences (CMHS), United Arab Emirates University (UAEU), P.O. Box 17666, Al Ain, United Arab Emirates; lizna@uaeu.ac.ae; 2 Zayed Bin Sultan Center for Health Sciences, CMHS, UAEU, P.O. Box 17666, Al Ain, United Arab Emirates; 3 Department of Biochemistry, CMHS, UAEU, P.O. Box 17666, Al Ain, United Arab Emirates

**Keywords:** retroviruses, RNA packaging, cross-/co-packaging, genomic RNA, psi, packaging signal, Gag proteins, nucleocapsid (NC), dimerization, recombination, viral variants

## Abstract

Retroviruses belong to the family *Retroviridae* and are ribonucleoprotein (RNP) particles that contain a dimeric RNA genome. Retroviral particle assembly is a complex process, and how the virus is able to recognize and specifically capture the genomic RNA (gRNA) among millions of other cellular and spliced retroviral RNAs has been the subject of extensive investigation over the last two decades. The specificity towards RNA packaging requires higher order interactions of the retroviral gRNA with the structural Gag proteins. Moreover, several retroviruses have been shown to have the ability to cross-/co-package gRNA from other retroviruses, despite little sequence homology. This review will compare the determinants of gRNA encapsidation among different retroviruses, followed by an examination of our current understanding of the interaction between diverse viral genomes and heterologous proteins, leading to their cross-/co-packaging. Retroviruses are well-known serious animal and human pathogens, and such a cross-/co-packaging phenomenon could result in the generation of novel viral variants with unknown pathogenic potential. At the same time, however, an enhanced understanding of the molecular mechanisms involved in these specific interactions makes retroviruses an attractive target for anti-viral drugs, vaccines, and vectors for human gene therapy.

## 1. Introduction

Retroviruses are a special a category of viruses that contain two copies of unspliced full-length genomic RNA (gRNA) as their genome rather than DNA. Similar to eukaryotic cellular RNAs, retroviral gRNA is a 7-to-10-kb terminally redundant molecule that contains a 5’ guanosine cap and a 3’ polyadenosine (poly(A)) tail [1] that is packaged as an RNA dimer [2,3]. The efficient and specific packaging of two copies of plus strand full-length gRNA into the virus particle from a large pool of cellular and other viral RNAs in the cytoplasm by the assembling virion is considered one of the hallmarks of retroviral life cycle (reviewed in [3,4,5,6,7,8,9,10]). This process of specific encapsidation involves the recognition of particular sequences known as the packaging signal (psi, Ψ, or E) by the zinc finger domain of the nucleocapsid (NC) binding site of Gag polyprotein (reviewed in [3,5,6,8,10]). Following dimerization of two strands of RNA genome close to their 5’ ends, retroviral RNA genome is packaged as a non-covalently linked dimer; therefore, both the process of RNA dimerization and that of RNA packaging are thought to be closely interlinked ([2] and reviewed in [3,5,6,7,8,11,12,13,14,15,16,17,18,19,20]). Consistent with this, for almost all retroviruses, determinants of RNA packaging and dimerization map to the same 5’ end region (100–400 nucleotides; nts) of the gRNA (reviewed in [3,5,6,21]). The only exception seems to be in human foamy viruses (HFV), where RNA dimerization does not seem to be a prerequisite for packaging [22].

Although gRNA packaging is a critical step of the fidelity of retroviral particle assembly, it can lead to cross-/co-packaging of heterologous gRNAs expressed endogenously or exogenously following infection of the same cell by two different retroviruses. This is despite the fact no sequence conservation between the packaging signals of different retroviruses has been found. Rather, it has been shown that the packaging sequences of all known retroviruses assume a higher order structure comprising different structural motifs (reviewed in [3,5,6,8,23]). Thus, regardless of their primary sequence, these structural motifs have been strongly associated with retroviral RNA encapsidation and could explain the phenomenon of RNA cross- and co-packaging among diverse retroviruses [7,24,25,26,27,28,29,30,31]. This review summarizes the current understanding of the gRNA encapsidation determinants of a diverse group of retroviruses, elaborates on what is known about the interaction of these structural elements with their cognate structural Gag proteins, and then discusses the current understanding of cross-/co-packaging among these retroviruses. Cross-packaging refers to the ability of structural proteins of one virus to recognize and encapsidate the gRNA from another virus, while co-packaging refers to the ability of two RNAs from distinct retroviruses to be packaged together into one virus particle. A better understanding of the phenomena of cross-/co-packaging among retroviruses should provide not only insights into the characterization of cardinal packaging determinants both at the RNA and protein levels among retroviruses, but also the possibility of generating novel recombinant viruses with unknown pathogenic potential. Moreover, it should also help improve the design and safety of retroviral vectors for human gene therapy.

## 2. How Similar Is the Structural Organization of Packaging Elements within Various Retroviruses?

**Figure 1:**
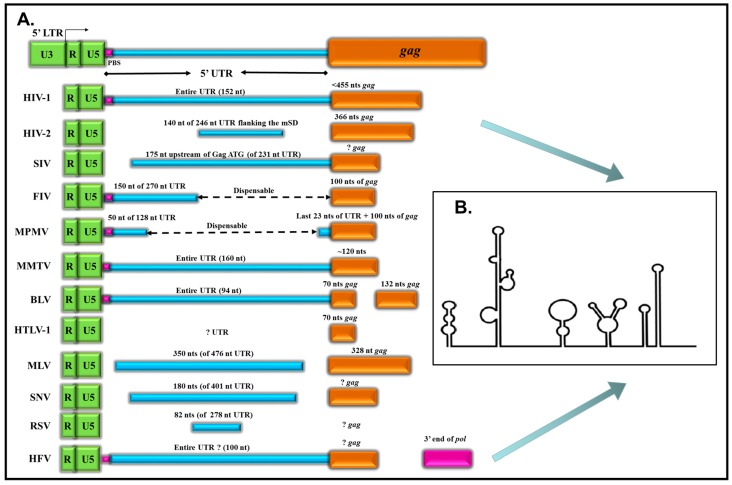
The primary packaging determinants of retroviruses are located at the 5’ end of the viral genome, within the 5’ untranslated region (UTR) and 5’ end of *gag*. (**A**) Schematic representation of the location of the packaging determinants within the 5’ UTR and *gag* for different retroviruses; (**B**) Generic representation of the higher order structures (stem loops) predicted, biochemically validated, or both at the 5’ end of different retroviruses important for gRNA dimerization and encapsidation. For the sake of simplicity and distinction from long terminal repeats (LTR) sequences, the 5’ UTR in this figure and Table 1 refers to sequences starting from the end of 5’ LTR (and not R) to the beginning of *gag* gene (excluding the ATG). Figure not drawn to scale. HIV: human immunodeficiency virus; SIV: simian immunodeficiency virus; FIV: feline immunodeficiency virus; MPMV: Mason-Pfizer monkey virus; MMTV: mouse mammary tumor virus; BLV: bovine leukemia virus; HTLV-1: human T-lymphotropic virus type 1; MLV: murine leukemia virus; SNV: avian spleen necrosis virus; RSV: Rous sarcoma virus; HFV: human foamy viruses.

The packaging signals have been identified and characterized for a number of simple and complex retroviruses using deletion and substitution analyses. They are generally located between the primer binding site (PBS) and the initial part of the *gag* gene (reviewed in [5,6,32,33]). Figure 1 and Table 1 summarize the location of the primary packaging determinants of several genus of retroviruses, from the simple alpharetroviruses to the complex lentiviruses and the eccentric spumaviruses. As can be observed, the packaging determinants can either encompass the entire 5’ untranslated region (5’ UTR), as illustrated for human immunodeficiency virus type 1 (HIV-1), mouse mammary tumor virus (MMTV), bovine leukemia virus (BLV), and HFV, or part thereof, as is the case for human immunodeficiency virus type 2 (HIV-2), feline immunodeficiency virus (FIV), murine leukemia virus (MLV), and Rous sarcoma virus (RSV). For most retroviruses, the proximal region (100–400 nts) of the *gag* gene is important in this process. In some cases, other regions of the viral genome have also been implicated such as the *pol* in the case of HFV [34,35,36], *env* in the case of RSV [37], or the 3’ UTR in the case of avian leucosis viruses and MLV [38,39].

Furthermore, the packaging determinants can either be continuous or discontinuous at the primary sequence level with regions in between not playing any seminal role in gRNA packaging. The bipartite or multipartite nature of packaging determinants has been observed for several retroviruses, including FIV, Mason-Pfizer monkey virus (MPMV), BLV, and HFV (Figure 1 and Table 1). These observations suggest that gRNA packaging involves RNA-protein interactions at a structural level that brings together non-contiguous regions of the viral genome architecturally in one place to facilitate the process of gRNA packaging. The sequences within the 5’ UTR have been thought to contain *cis*-acting sequences important for protein binding (Gag) and dimerization initiation, while sequences in the *gag* gene have been speculated to stabilize the secondary structure of the region and facilitate the process of gRNA packaging into the assembling virions.

The first demonstration that *cis*-acting structural elements may be important for RNA packaging came with the observation that two hairpins were found to be involved in the packaging of the avian spleen necrosis virus (SNV) gRNA [58,62]. Interestingly, these structures could be replaced functionally with those from Moloney murine leukemia and murine sarcoma viruses with no effect on the replication of SNV-based vectors [58]. Thus, this was not only the first report of the importance of structural elements for RNA packaging, but also of cross-packaging among retroviruses. Since then, RNA folding algorithms, phylogenetic analyses, and biochemical probing have all revealed that the 5’ end of the retroviral genome folds into complex RNA secondary structures (stem loops) that affect retroviral gRNA packaging for retroviruses at biological levels. These structures contain helix-loop motifs with terminal purine-rich loops that specifically interact with the nucleocapsid protein to allow specific gRNA encapsidation into the viral particles.

The packaging determinants of HIV-1 and MLV have been the most extensively studied (reviewed in [5,8,33]). In HIV-1 and MLV, these regions are found predominantly downstream of the major splice donor (SD) in the 5’ UTR [54,55,63,64,65], and the *gag*-coding region [55,66,67]. However, in HIV-1, sequences upstream of the SD are also important [14,68]. The characteristic stem loops observed at the 5’ end of the HIV-1 genome correspond to important steps of the virus replication cycle that are either virus-specific (such as the *trans*-activation response element (TAR) loop important for transcriptional transactivation by the HIV-1 Tat protein), or more generic (such as the poly(A), U5/PBS, dimer initiation site (DIS), and psi stem loops) common to many retroviruses with critical roles both at the primary and structural levels. For example, both the sequence of DIS (CGGCCG and CUGCAG) and its location on a stem loop structure is important for gRNA dimerization in MPMV and MMTV, respectively [69,70]. The structural motifs at the 5’ end of the gRNA also interact at tertiary levels with each other and other parts of the viral genome for proper function. For example, the poly(A) stem loop of HIV-1 is involved in long-range interactions (LRIs) with sequences in the matrix (MA), forming a pseudoknot [71]. Similarly, the U5 stem loop sequences are involved in long-range interactions (LRI) with the stem loop containing the Gag AUG in many retroviruses, including HIV-1 and 2, SIV, FIV, MMTV, and MPMV [69,70,71,72,73,74,75,76,77,78,79], as well as other plus-strand RNA viruses with icosahedral capsids [80]. Mutations that destabilize these interactions affect several important steps in the retroviral replication cycle, including RNA packaging and dimerization [10,48,49,71,72,79,81,82].

Detailed RNA structural determinants of two important beta retroviruses, MMTV and MPMV, as well as lentiviruses such as HIV-1 and FIV have recently been reported as employing selective 2’-hydroxyl acylation analyzed by primer extension (SHAPE) followed by validation of predicted structural motifs for RNA packaging and dimerization using mutational analysis in a biologically relevant replication assay [69,70,74,76,79,82,83]. Emerging themes from these and other studies (reviewed in [3,8,33]) on RNA packaging can be summarized as follows:

1. The 5’ ends of retroviral genomes, starting from R to 100–400 nts into the *gag* gene fold into several stable, but flexible, helix-loop motifs termed stem loops (SLs) with purine-rich loops important for protein binding (Figure 1).

2. The RNA structure is held together by LRIs between the U5 and Gag sequences that occlude Gag AUG. Disruption of the LRIs affects both RNA dimerization and packaging.

3. Dimerization is thought to be a prerequisite for RNA packaging and is initiated by a GC-rich palindromic DIS containing a canonical GC dyad that is present in close proximity or overlapping the packaging determinants.

4. The DIS itself can be found in both “occluded” and “accessible” RNA conformations, allowing the cells to regulate ratios of monomeric and dimeric forms of RNA, the former of which is suited for translation (Gag AUG-accessible), while the latter for RNA packaging (Gag AUG-occluded).

5. Initiation of genomic RNA dimerization takes place via “kissing-loop” interactions through DIS on two RNA molecules (intermolecular interactions), which leads to the formation of the dimer linkage structure (DLS) that allows for the differentiation of gRNAs from mRNAs.

6. Furthermore, these conformational changes or “switches” result in the exposure of the otherwise hidden or obscured purine-rich nucleocapsid binding sites on the RNA (single-stranded (ss)purines-sspurines), initiating the process of Gag binding via the NC domain and multimerization on the RNA, leading to RNA encapsidation and virion assembly.

7. Depending upon the virus, the recruitment of dimerized gRNA by Gag takes place either in the nucleus (RSV and MLV), the pericentriolar region (MMTV and MPMV), the nuclear membrane (FIV), or the cytoplasm (HIV-1), followed by virion assembly either in the cytoplasm or the plasma membrane.

An interesting spatial feature observed among packaging signals of different retroviruses is the relative distance between the packaging signal and the 5’ methyl cap of the gRNA. This distance appears to be ~300–400 nt long at the 3’ end of the cap site for a number of simple and complex retroviruses, including SNV, RSV, MLV, MPMV, HIV-1, HIV-2, and SIV [84,85,86]. This distance is speculated to be critical for the encapsidation function and may relate to the competition between translation and encapsidation functions of the gRNA, which are necessary to ensure optimal replication efficiency of the virus [86]. How retroviruses maintain pools of gRNA to be used for Gag/Pol protein translation and as substrates for RNA packaging is not entirely clear, but the emerging picture suggests a role of internal ribosome binding sites (IRES) present within the 5’ UTR as well as within coding sequences of the genome that allows for the translation of Gag, independent of cap-dependent translation [87]. Having multiple mechanisms to ensure structural protein synthesis may be important for RNA packaging and virus assembly. This may also have relevance for the co-translational mechanism of gRNA packaging proposed for some retroviruses, where the primary packaging determinant is found upstream of the major splice donor and thus present on both genomic and spliced RNAs, such as RSV [59,88] and HIV-2 [42]. Accordingly, low amounts of Gag stimulate translation of Gag proteins from the full-length viral RNA in a cap-dependent manner. An increasing amount of Gag concentration in the cell then stimulates binding to the packaging signal at the 5’ UTR/beginning of *gag* in a co-translational manner [42,89,90,91]. Conformational changes then lead to multimerization of the protein on the gRNA scaffold, causing repression of translation from the cap structure while allowing protein synthesis from the IRES, thus stimulating RNA packaging and virus assembly [87].

Finally, the mechanism behind how viral genomic RNA is packaged preferentially to that of spliced viral RNAs in retroviruses where the primary packaging determinants are present in both spliced and unspliced RNAs is not entirely clear. It has been observed that, for HIV-1 and RSV, up to 10% of the packaged RNA may be spliced [59,92]. In the case of RSV, Env mRNA is the only spliced message of the virus translated on endoplasmic reticulum (ER)-associated ribosomes. Thus, the translated signal peptide sequesters the complex into the rough endoplasmic reticulum away from the Gag protein. The Gag protein, on the other hand, is produced on free cytoplasmic ribosomes, ready for binding to gRNA for virion assembly in a co-translational manner [89,90]. The spatial separation of the two mRNAs thus allows for selection for the genomic and not the spliced mRNA for packaging in RSV. Similarly, for HIV-2, specificity may be maintained by co-translational packaging of the Gag-encoding unspliced message [42,91], which also limits the amount of Gag available that could capture other spliced mRNAs [42].

## 3. What Determines the Specificity of gRNA Packaging among Retroviruses?

**Figure 2:**
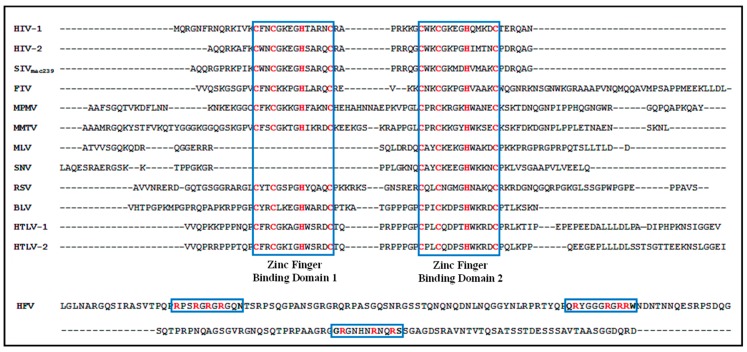
Multiple sequence alignment of the nucleocapsid region of the various Gag proteins of the listed retroviruses. The zinc finger binding domains present in the protein are boxed. The conserved cysteine (C) and histidine (H) amino acids of the zinc finger domains are shown in red. As can be observed, MLV and SNV contain only one zinc finger binding domain. The spumaviruses (e.g., HFV) are unique among retroviruses in that they contain three glycine-arginine-rich boxes instead of the cysteine-histidine-containing zinc finger domains [32]. Dashes have been added to facilitate the alignment of the differently sized nucleocapsid proteins.

The packaging determinants of retroviral gRNA interact with the highly basic NC domain of the Gag precursor polyprotein to capture the gRNA for packaging into the assembling virus particles [3,5,6,8,11,21,32,83]. Elimination of the NC domain from Gag disrupts the specific packaging of HIV-1 gRNA, while packaging of cellular RNAs is supported by basic residues in the MA domain of Gag [1,93,94]. Depending upon the viral strain analyzed, NC contains either one to two evolutionarily conserved, but distinct, Cys-His boxes [95,96] that can sequester Zn^2+^ ions, allowing for high affinity NC-gRNA interactions [97,98,99,100,101]. Spumaviruses are an exception to this model in that that they contain arginine-rich motifs instead of the zinc fingers. The Cys-His boxes contain conserved CCHC arrays (C-X2-C-X4-H-X4-C where C = Cys, H = His, Xn = n number of amino acids) that are variable between different retroviruses (Figure 2). Flanking (and sometimes overlapping) the Cys-His boxes are stretches of basic amino acids important for specific RNA binding of NC [102] that are found in many retroviruses. Mutations in the conserved CCHC boxes can significantly reduce genomic RNA packaging and give rise to non-infectious virions [65,102,103,104,105,106,107,108,109,110,111,112,113,114,115]. Mutations in the flanking regions of Cys-His boxes, which contain basic residues, also result in severe replication defects [102,116,117,118]. Furthermore, studies with chimeric Gag proteins have revealed that specificity of RNA packaging could be interchanged when the NC domains between two viruses were switched, such as those between MLV and RSV [119] and between MLV and HIV-1 [110,120]. Since the amino acid sequence of the regions flanking the Cys-His boxes are quite diverse (see Figure 2), the flanking regions of the NCs were shown to be “functionally conserved” by replacing a flanking region from SNV with that of MLV and vice versa, which did not affect either Gag polyprotein or NC function for virus RNA encapsidation or virion assembly [121]. These studies imply that NC and its flanking regions are a critical component determining specificity of gRNA packaging, though they may not be the sole determinant (see below).

The zinc finger domains of HIV-1 NC bind specifically to the viral genomic RNA via the encapsidation signal [122]. The two zinc fingers are not functionally equivalent or interchangeable as evidenced by experiments in which the two motifs were either switched or duplicated [108]: the first Cys-His box plays a more prominent role in encapsidation and is more selective for genomic viral RNA for encapsidation. This is evidenced by the fact that mutations in the first Cys-His box have a more drastic effect on gRNA packaging (100-fold) than those in the second box (10–30-fold) [105,106]. Extensive structural analysis of NC-psi sequences has been carried out using nuclear magnetic resonance (NMR) studies defining the actual molecular interactions that take place between NC and the packaging signals (reviewed in [8]). NC probably binds to dimeric genomes, and this interaction leads to the multimerization of the Gag polyproteins, leading to virus assembly [123,124].

Despite the fact that NC has been shown to be important for gRNA packaging, several lines of evidence suggest that NC may not be the only determinant of specific gRNA packaging, and other Gag domains may also be involved. For one, NC is a highly basic protein; as such, it has non-specific RNA binding activity [120]. Furthermore, RNA packaging experiments using chimeric Gag proteins between HIV-1 and MMTV where the NC domains of the respective viruses were swapped revealed that these viruses still packaged their cognate gRNAs preferentially [125], unlike what was observed for chimeras between MLV and RSV and between MLV and HIV-1 discussed above. Moreover, several independent studies have implicated other regions of the Gag polyprotein, including MA [10], capsid (CA) [126], the p2 spacer peptide between CA and NC [40,127,128], and the terminal p6 late domain [110]. Considering that the Gag polyprotein is the initial recruiter of gRNA and not the cleaved NC protein, this suggests that specific selection of gRNA from other cellular and spliced RNAs is more complex and happens in the context of the whole polyprotein, as has recently been shown for HIV-1 [83].

The intracellular sites of initial interaction between gRNA and NC domain of the Gag polyprotein also have important implications for virion RNA packaging and assembly. These interactions can occur in the nucleus or the cytoplasm, depending upon the nature of the retrovirus [33]. The simple retroviruses such as RSV and MLV use the help of the Gag proteins containing nuclear localization signals and the cellular importin-α/β proteins to enter the nucleus to export the gRNA to the cytoplasm [129]. In these viruses, the gRNA is thought to dimerize in the nucleus before interactions of the packaging signal with the NC domain of the viral Gag polyprotein [130]. This gRNA/Gag complex is then transported into the cytoplasm via the cellular chromosome region maintenance 1 (CRM1) RNA export pathway for the eventual virus assembly at the plasma membrane [131]. The complex retroviruses such as HIV-1 and FIV, on the other hand, can export the genomic unspliced RNA using their accessory genes (*rev*) involved in the nucleocytoplasmic passage of unspliced, full-length gRNA, which would otherwise get spliced by the cellular splicing machinery [132]. Thus, for these viruses, the gRNA exits the nucleus and first interacts with the Gag proteins in the cytoplasm, either at the nuclear membrane, as is the case with FIV, or the cytosol, as is the case with HIV [133,134]. Either way, the gRNA/Gag polyprotein complexes are then directed to the cell membrane for final assembly into the virus particles. This process employs the matrix domain of the Gag polyprotein that not only has the *N*-myristoyl moiety for plasma membrane targeting, but also a stretch of basic amino acids that can interact with both gRNA and plasma membrane-specific phospholipids for proper targeting [135,136].

## 4. How Do Retroviruses Cross-Package Each Other’s Genomes?

Based on the evidence presented above, it is clear that the packaging determinants of retroviruses at the three-dimensional RNA structural level are spread out and elastic, composed of primary, secondary, as well as tertiary interactions. This lack of rigidity and absolute specificity is necessary for the numerous conformational changes that need to take place to allow successful packaging of the gRNA preferentially over the spliced as well as the cellular mRNAs. A direct consequence of this flexibility is RNA pseudotyping or cross-packaging; i.e., packaging of an RNA by heterologous viral particles that can take place among evolutionarily related yet molecularly different retroviruses [24,25,28,29,30,31,40,58,137,138,139,140,141]. Furthermore, co-packaging of heterodimeric RNAs from two divergent retroviruses can also take place [26,27,31], resulting in recombinant viruses with unknown pathogenic potential, if successful reverse transcription and template switching can take place following the co-packaging of two genetically distinct RNAs.

Thus, cross- and co-packaging among retroviruses has been investigated to determine the potential hazards and safety of using retroviral-based vectors for human gene therapy since patients being treated could be infected by other retroviruses, while the human genome itself contains endogenous retroviruses that could provide substrates for cross- or co-packaging [142,143,144,145,146,147]. Furthermore, a better understanding of these phenomena should help in the development of novel retroviral vectors (both recombinant/hybrid and otherwise) for safer and more efficient gene delivery systems into humans [148,149,150,151,152,153,154]. Towards this end, genomes of several different retroviruses from different hosts have been explored, including the simian lentivirus SIV [30,137,155] the feline lentivirus FIV [156,157], the avian retrovirus SNV [28], the mouse retroviruses MLV and MMTV [158,159], and even the more simian-like human lentivirus, HIV-2 [137]. These studies have shown that it is possible to develop novel hybrid vector systems (e.g., SNV/HIV-1 [28] and SIV/HIV-2 [137]) that maintain high transduction efficiencies with possibly reduced pathogenic potential.

One of the earliest examples of cross-packaging among retroviruses was observed between the avian SNV and murine MLV retroviruses when it was shown in a series of elegant studies that a SNV helper cell line producing SNV structural proteins could cross- and co-package RNA from the distantly related retrovirus, MLV, and generate recombinants [31,58,141,160]. Since then, a number of human and animal retroviruses have been studied for their cross-packaging potential using genetic complementation and/or biological replication assays. For example, among complex retroviruses, several studies have revealed that lentiviruses such as HIV-1, SIV, and even the more distantly related FIV, can cross-package each other’s genomes [29,30,161]. Subsequently, these observations were expanded to the beta retroviruses, when reciprocal cross-packaging between the two distantly related type B and D retroviruses—MMTV and MPMV respectively—was reported [25]. Not only that, reciprocal cross-packaging has also been demonstrated between HIV-1 and SIV and between MPMV RNA and proteins [24]. In most of these cases, cross-packaging was demonstrated to be primarily due to the recognition of the packaging signal by the heterologous proteins.

In addition to reciprocal cross-packaging, non-reciprocal packaging has also been observed, between simple retroviruses where the SNV genome could not be cross-packaged by MLV proteins [58,138,141,160]. Moreover, RNA from a complex retrovirus (HIV-1) could be cross-packaged by proteins from a simple retrovirus, SNV, but not vice versa [28]. On the contrary, RNA from a complex retrovirus (FIV) could not be cross-packaged by proteins from a simple retrovirus (MPMV), while FIV proteins were efficient at cross-packaging MPMV RNA [161].

Non-reciprocal cross-packaging has also been observed even among more closely related retroviruses. For example, the human lentivirus HIV-2 that can also cause acquired immunodeficiency syndrome (AIDS) like HIV-1, although to a much more restricted extent, is phylogenetically closer to SIVs than to HIV-1. Interestingly, HIV-2 RNA has been shown to be cross-packaged by HIV-1 proteins, but HIV-2 proteins were unable to encapsidate and transduce HIV-1 RNA [40,137]. However, HIV-2 Gag chimeras that contained the HIV-1 NC and p2 domains were able to encapsidate HIV-1 RNA. HIV-2 Gag chimeras with only the HIV-1 NC domain also exhibited the ability to encapsidate HIV-1 RNA, although at a lower level than when both NC and p2 were present, suggesting that amino acids within the p2 domain also contribute to RNA packaging. The HIV-1 and HIV-2 NC proteins are quite similar with 60% homology at the amino acid level and only conservative amino acid difference in other parts of the protein, while the p2 domain is much less similar with only 35% homology at the amino acid levels. This supports the hypothesis that selective recognition may be spread over other regions of the Gag precursor [40,137]. One of the reasons for the observed packaging restriction could be the co-translational mode of packaging observed in HIV-2, which is distinct from the *trans*-packaging observed in HIV-1 [91]. Replacement of HIV-2 NC with that of HIV-1 could have relieved that inhibition to allow cross-packaging perhaps by using the co-translational mode of packaging.

Table 2 summarizes many of the studies available in literature regarding cross-packaging of retroviral genomes and their subsequent transduction (propagation) into target cells. As can be seen, with the exception of HIV-2, the other lentiviral proteins tested (HIV-1, SIV, and FIV) were the most promiscuous for allowing RNA cross-packaging, as they could not only cross-package each other’s genomes, but also genomes from MPMV, MMTV, and MLV (tested only for HIV-1). On the other hand, the HIV-2 and MLV proteins seemed the least promiscuous, unable to cross-package RNA from any of the tested viruses. HIV-2 could neither package HIV-1 nor SIV RNAs despite close genetic relationship with these viruses. Similarly, MLV proteins could neither package SNV RNA (an avian virus that is closer to the mouse retroviruses than other avian viruses), or RNAs from other more distantly related retroviruses such as HIV-1 or MMTV. On the contrary, SNV proteins could cross-package RNAs from both MLV and the distantly related HIV-1.

Interestingly, even though HIV-1 proteins were the most promiscuous among the retroviruses tested, they were unable to cross-package SNV RNA. Among the beta retroviruses, both MPMV and MMTV proteins could cross-package RNAs from the tested heterologous viral RNAs with the exception of FIV RNA that could not be cross-packaged by MPMV proteins. This is despite the fact MPMV could cross-package both HIV-1 and SIV RNAs. The inability of SNV RNA to be cross-packaged by either HIV-1 or chimeric MLV NC proteins or the FIV RNA by MPMV proteins could probably be explained by restrictions perhaps not at the RNA end, but at the protein level, such as the NC and matrix proteins as has been suggested earlier for some retroviruses [110,125]. Finally, the cross-packaging of MLV RNA has also been observed by both RSV and BLV proteins in addition to HIV-1, even though phylogenetically MLV RNA is far from these viruses.

Thus, the ability to cross- or co-package a retroviral genomic RNA into heterologous structural proteins is a lot more complex and involved. It depends upon not only the packaging determinants on the gRNA and the zinc finger domains of the NC protein, but also by the type of viral RNA available for packaging, its cellular location (nuclear versus cytoplasmic), mechanistic differences in packaging (*cis* versus *trans*), the role of Gag domains other than NC, etc. Considering that gRNA in the virion does not exist as a linear, dimeric species but rather in a highly compact and condensed form as a nucleoprotein complex [162] adds another layer of complexity to this phenomena, making empirical testing still the best way to determine whether two viral genomes can successfully cross- or co-package, or both, their gRNAs into a particular virus particle.

## 5. What Determines the Propagation Capabilities of the Cross-Packaged RNAs?

Table 2 also summarizes the capability of different retroviruses to propagate (transduce) the cross-packaged RNAs by the heterologous viral proteins. As can be seen, the propagation of the cross-packaged RNA into the target cells has been found to be quite frequent among retroviruses, though to a lesser extent than RNA cross-packaging. This suggests that the successful propagation of the cross-packaged RNA must involve compatible interactions between the *cis*- and *trans*-acting sequences needed for reverse transcription and integration. This includes the ability of the reverse transcriptase enzyme of the packaging viral particles to recognize the PBS and polypurine tracts (PPT), the viral integrase to recognize the attachment (att) sites of the cross-packaged RNA, or both.

**Figure 3:**
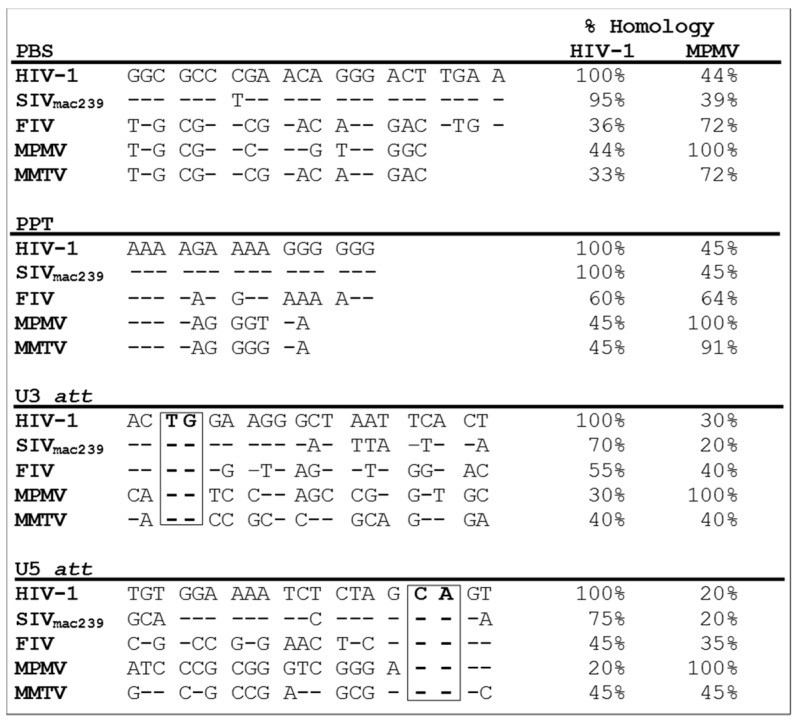
Comparison of the major *cis*-acting sequences among different retroviruses required for the reverse transcription and integration steps of retroviral life cycle. The canonical TG and CA dinucleotides within the 3’ and 5’ attachment (att) sequences, respectively, are boxed. All sequences are compared to either that of HIV-1 or MPMV, where a “-“ represents homology, while differences are shown by the native nucleotide sequence. PBS: primer binding site; PPT: polypurine tract; U3 att: 3’ attachment site in the U3 region; U5 att: 5’ attachment site in the U5 region of the long terminal repeat.

Overall, Table 2 shows that, if a viral RNA could be cross-packaged, nearly 60% of the time it could also successfully propagate and transduce the target cells. However, there were exceptions. For example, the most promiscuous lentiviruses, HIV-1, SIV, and FIV, could cross-package each other’s RNAs and propagate them successfully; they could also cross-package MPMV (a non-lentivirus) RNA as well, but all three lentiviruses were unable to propagate the cross-packaged MPMV RNA into the target cells [24,161]. Similarly, MPMV proteins could cross-package HIV-1 and SIV RNAs (though not FIV), but could not propagate these RNAs further, pointing towards the incompatibility of the MPMV reverse transcriptase and integrase enzymes with the *cis*-acting sequences of the heterologous (HIV-1 and SIV) viral RNAs. A comparison of the *cis*-acting sequences important for viral RNA propagation among HIV-1, SIV, FIV, and MPMV revealed size differences in the length of PBS and PPT (shorter for MPMV than the lentiviruses) as well as the least homology of the MPMV att sites compared to HIV-1, SIV, or FIV (Figure 3). These observations suggest that incompatibility between FIV reverse transcriptase and integrase enzymes with that of MPMV PBS, PPT, and att sites is the likely reason for the inefficient RNA propagation observed between MPMV and the lentiviruses. Similar observations have been reported when HIV-1 att sites were substituted by those from FIV in HIV-1 RNA, resulting in only a one-third reduction in integration efficiency, while substitution with the more distantly related MLV att sites led to nearly a complete abrogation of integration of the hybrid HIV-1 RNA [164].

As can be further observed from Table 2, HIV-2 and MLV were the two viruses that were the most restrictive in their ability to cross-package heterologous RNAs. SNV, on the other hand, was more like HIV-1, SIV, and FIV, and could not only cross-package HIV-1 and MLV RNAs, but also successfully propagate these RNAs into the target cells, suggesting compatibility of its reverse transcriptase and integrase enzymes for the *cis*-acting sequences of the heterologous viruses.

As mentioned earlier, the two type B and D betaretroviruses—MMTV and MPMV—could reciprocally cross-package their genomes; however, both viral proteins were unable to transduce the infected cells with each other’s cross-packaged RNAs [25]. Sequence analysis of the *cis*-acting elements of MMTV and MPMV RNAs revealed that MMTV PBS had a 72% sequence homology to MPMV, while MMTV PPT showed a 90% homology (Figure 3). However, comparison of att sites indicated a sequence homology of less than 50% (the U3 att was 40%, whereas the U5 att was 45%), suggesting problems with reverse transcription and integration. Thus, the sequences of RNase H and integrase enzymes were also compared, which were found to be only 32% and 49% homologous, respectively [25]. Based on these analyses and the fact that both of these viruses utilize tRNA^Lys^ primers (Lys-1 and -2 for MPMV and Lys-3 for MMTV), it was proposed that these viruses probably could not transduce the cross-packaged RNAs either due to inadequate reverse transcription or integration of fully reverse-transcribed DNA, or both, resulting in failed propagation [25]. Several studies support these assertions [24,28,161,164,165]. In a study by Parveen et al. [28], the role of integrase in improving transduction efficiency of cross-packaged HIV-1 RNAs by SNV proteins was studied in particular. This was achieved by making chimeric Gag/Pol packaging constructs where different domains of SNV integrase were replaced by that of HIV-1. Only one of the chimeric integrase (IN) proteins was able to transduce the HIV-1 vector RNA, albeit at the same efficiency as the wild-type SNV packaging construct [28]. This study suggests that efficient propagation of cross-packaged RNAs requires overcoming multiple post-entry blocks of the virus replication cycle.

## 6. Packaging of Non-Viral RNAs into Retroviral Particles

How can one be assured that, once cross-packaging is observed, it is due indeed to the specific recognition of the packaging determinants and not due to either the known non-specific nucleic acid binding activity of NC [21] or other artifacts of the experimental system such as overexpression of the expressed RNA to be packaged into the cell? One way to demonstrate specificity of a newly mapped packaging signal is to clone the packaging signal in question onto a non-viral RNA and determine if that RNA can then be packaged specifically by the homologous protein. This approach has been used to confirm specificity of RNA packaging for a number of retroviruses, including MLV [55], BLV [51], and FIV [166]. The same approach was used to demonstrate specificity of cross-packaging between MPMV and MMTV [25], and that between HIV-1, SIV, and MPMV [24,25] as well. Thus, the cloning of the putative packaging determinants of MPMV and MMTV into pcDNA3-based vector RNAs confirmed that these non-viral RNAs could be co- and cross-packaged by the homologous and heterologous proteins [24,25]. To ensure that the results obtained were not due to overexpression of the vector RNAs, control vector RNAs using the same RNAs were expressed, but in the absence of the packaging signals. Together, these results reveal that neither the non-specific binding affinity of NC for RNA nor overexpression of the vectors is responsible for the cross-packaging activities being observed.

## 7. Packaging of Cellular RNAs into Retroviral Particles

It has been estimated that gRNA dimer is the dominant RNA species found in a virus particle accounting for ~50% [21] of the virion RNA content by weight along with 10–50 tRNA molecules used by the virus to prime reverse transcription [1]. The question that naturally arises is: what other RNAs are packaged into the virus particles, and are they packaged specifically? It has been shown that, other than gRNA, other cellular [21,167,168,169,170,171,172,173] and occasionally spliced viral RNAs [59,92,174,175,176] are also packaged. Packaging of the spliced viral mRNAs is no surprise since, depending upon the virus, the primary or secondary packaging determinants may be found upstream of the SD site; hence, these signals could be present in both the spliced and unspliced viral mRNAs, allowing their specific or semi-specific packaging into the virus particles. However, the role that these spliced viral mRNAs might play in the virus life cycle is not clear.

What about the cellular RNAs and their packaging into the virus particles? Cellular RNAs represent the remaining 50% of the virion RNA content in the same proportion as observed in the cells [1,177]. Since most of the cellular RNAs are composed of non-coding RNAs (ncRNAs), these are the types of RNAs also found in virions (reviewed in [178]). Among these, some have been observed to be enriched in virus particles such as the 7SL RNA, 5S rRNA, the spliceosomal U6 small nuclear RNA (snRNA), cytoplasmic Y RNA, and some cellular tRNAs [178]. On the contrary, no specific cellular mRNA has been found to be enriched in the virus particles. The mechanism of how the ncRNAs may be encapsidated into the virus particles is still under investigation and may require both specific and non-specific interactions with regions within Gag, Pol, or even the gRNA itself [178]. The discovery that some non-coding RNAs may be specifically packaged by the virions suggests novel ways of how retroviruses might use ncRNAs in their assembly and infectivity processes.

## 8. Co-packaging and Consequences of Co-Packaging among Diverse Retroviruses

All retroviruses encapsidate two copies of full-length gRNAs as a non-covalently linked dimer into the virus particles. Co-packaging is thus a natural part of the retroviral life cycle during which retroviruses package their gRNAs in pairs, even when the cells have limiting amounts of gRNA [179]. The norm is that the two gRNA copies are identical when a cell is infected with one type of virus, resulting in homodimers. However, if a cell is infected with more than one virus, or contains endogenous viruses that express viral RNA with compatible DIS, a heterodimer may form that could then be packaged by the expressed Gag proteins into retroviral particles. Co-infection with divergent retroviruses or co-transfection of experimentally produced retroviral vectors have been shown to result in the co-packaging of two different retroviral RNAs within a virus particle. For example, HIV-1 RNA can heterodimerize with either MLV or RSV RNAs [180], while MLV can co-package SNV [31]. A direct consequence of co-packaging is the exchange of genetic information during reverse transcription resulting in the formation of retroviral recombinants. For instance, the co-packaging and exchange of genetic information between genetically different retroviruses such as SNV and MLV [31] as well as between HIV-1 and HIV-2 have been reported [26]. The generation of viral variants can provide evolutionary advantages to the recombinants such as the acquisition of new or unknown pathogenic potential that may cause diseases, allow escape from the host’s immune system, or provide the recombinants with expanded host tropism [179,181,182].

### 8.1. Role of Dimerization in Co-Packaging

RNA dimerization allows for the formation of a unique RNA configuration—the dimer linkage structure—that helps distinguish gRNAs from mRNAs [3,179]. Other than ensuring specificity of gRNA packaging, dimerization is an essential prerequisite for the cross- and co-packaging of gRNAs. Packaging of dimeric RNAs is thought to increase the fidelity of genomic RNA replication, allowing retroviruses to generate full-length intact proviruses, even though persistent nicking of gRNA is observed [3,179], while co-packaging allows for virus evolution (increased genetic diversity) due to the generation of new recombinant proviruses. As discussed earlier, the efficiency of heterodimer formation is affected by the cellular site and timing of gRNA dimerization which has been shown to be different in different retroviruses (reviewed in [3,33]). As a result, different types of dimers are possible: viruses that dimerize late in the replication cycle such as in the cytoplasm (e.g., HIV-1 and FIV) have the possibility to form both homodimers as well as heterodimers, whereas viruses undergoing dimerization soon after transcription in the nucleus (such as MLV and RSV) primarily form homodimers even if other compatible RNAs are present. Thus, viruses such as HIV-1 that dimerize in the cytoplasm have a higher potential to co-package gRNAs as heterodimers [179,181].

The co-packaging frequency of the heterologous RNA can be influenced by their DIS sequences. When the two DIS sequences of HIV-1 and HIV-2 were mutated to simultaneously discourage RNA homodimerization and encourage RNA heterodimerization, HIV-1 and HIV-2 RNAs could heterodimerize using the DIS sequences prior to packaging [183,184]. Hence, it seems that the major determinant for co-packaging is to have a complementary DIS sequence among the two RNAs. Studies have shown that RNAs carrying palindromic or non-palindromic DIS sequence can heterodimerize if the DIS from two distinct viruses are complementary to each other [4,7,27,185,186]. Consistent with this, in vitro-transcribed trans-complementary mutants maintaining the central “GC” dyads have shown efficient RNA dimerization, authenticating that an intermolecular interaction mediated by the DIS is required for the formation of a stable RNA dimer [19,69].

### 8.2. Recombination Potential of Retroviruses

A salient characteristic of retrovirus replication is the high prevalence of genetic recombination that can assort mutations in the viral genome to increase replication fidelity and virus diversity. Recombinants are often generated after co-infection of a cell by two genetically distinct viruses, a feature first observed in avian tumor viruses [187] and later in other retroviruses as well [188,189,190]. The presence of multiple receptors on a target cell facilitates dual infection which is often cell-mediated [191,192,193]. The two distinct genomes need to be co-packaged, leading to heterozygous virion formation, for successful recombination to occur [194,195].

Recombination is a consequence of reverse transcription, a process that requires the reverse transcriptase (RT) to jump several times between the two genomic RNA templates to create a single DNA copy of the genome. It has been estimated that, during the process of reverse transcription, RT dissociates at least eight times for a 10-kb viral genome [196,197,198]. The template switching can be intermolecular [195,199,200,201], resulting in recombination [199,202,203,204,205]. It can also be intramolecular, resulting in deletions, deletions with insertions, or insertions and duplications [196,206,207], or it can be in a fashion that the first strand transfer step in reverse transcription is an intermolecular event while the second transfer is intramolecular [208]. Thus, generation of a recombinant virus requires multiple steps to happen successfully, such as (1) dual infection of a cell; (2) expression of both viral genomes; (3) dimerization of the heterologous RNAs; (4) successful co-packaging of the heterodimeric gRNAs; (4) RT template switching between the heteromeric co-packaged RNAs; and (5) integration and expression of the recombinant DNA. Despite this complexity, retroviruses still possess one of the highest recombination rates among viruses [181].

A comparison of sequences among different retroviruses suggests that ancient recombination events between different retroviruses may have occurred [2]. A classic example of this is the avian retrovirus SNV. The *gag/pol* region of SNV is related to that of MLV, whereas the *env* region is related to that of the simian virus MPMV [139]. This assertion is further supported by the observation that SNV and MPMV Env proteins bind to the same receptor during infection [139,209]. Another evidence for genetic exchange and recombination has been observed with the squirrel monkey retrovirus (SMRV), a type D retrovirus in which the *env* sequences were found to be homologous to the type C baboon endogenous retrovirus (BaEV), while the *pol* gene to type A, type B and avian type C retroviruses [210]. The LTRs sequences of SMRV were found to be present in another New World species: the skunk. However, the PBS was unique to type D retrovirus [210]. These kind of recombination events are likely to occur more than once in the evolution of retroviruses and therefore retroviruses are believed to have originated as a consequence of enormous genetic exchanges, following multiple contact or infections of the host with different species of retroviruses [211].

What are some of the elements that affect recombination frequency? Longer lengths of homology are one of the driving forces that influence a successful template switch [181]. Thus, recombinants between highly similar strains are generated at the highest frequencies than more diverse viruses [212,213]. Differences in site of dimerization of the two viral heterologous RNAs can also contribute to differences in recombination frequencies. For example, even though the RTs of HIV-1, SNV, and MLV appear to have similar processivity, the recombination rates of gammaretroviruses are 10-fold lower than those of lentiviruses [214,215]. This has now been attributed to the early commitment to dimerization of gammaretroviruses RNAs while still within the nucleus, and results in favoring homo- rather than heterodimers [179]. HIV-1 exports its gRNA into the cytoplasm before dimerization can take place. This later stage of commitment has been shown to result in the random distribution of heterodimers, which naturally result in higher frequencies of recombination. Similarly, the export pathway selected by the transcribed viral RNAs to exit the nucleus can affect its co-packaging [7]. Thus, two viruses that use the same export pathway for their RNA transport (e.g. nuclear RNA export factor 1 (NXF1) pathway) are also co-packaged more efficiently [7].

The secondary RNA structure of the gRNA also influences the recombination efficiency. Specific hairpin structures on the template RNA have been shown to stimulate RT for strand transfer to the acceptor DNA in the presence of NC protein [216]. In fact, different groups have identified recombination hotspots associated with RNA hairpins in the R region [217], *gag* [218], *pol* [219], and *env* [220,221]. Given the association between RNA stem-loops and recombination, these identified structures may facilitate recombination and regulate translation as well [181]. Similarly, the NC protein affects recombination efficiency by influencing various steps of viral replication. In addition to the already-discussed roles of NC in specific gRNA packaging and virus assembly, NC also affects tRNA placement onto the template RNA, dimerization, reverse transcription, and integration of the provirus [21,95,96,103,110,117,120,125,222,223,224,225,226].

## 9. Concluding Remarks

The ability of heterologous retroviruses to cross- and co-package their gRNAs once again reinforces the notion that retroviruses are highly promiscuous genetic mobile elements. This suggests that, if co-infected, co-packaged, and heterodimerized, these viruses could provide an enormous substrate for genetic recombination that might lead to the generation of replication-competent viral variants with unknown pathogenic potential, as well as drug- and vaccine-resistant viral variants. Therefore, delineating the requirements and factors that influence heterologous RNA cross- and co-packaging should help better understand the phenomenon of not only the packaging, but also the evolution of variant viruses with unknown pathogenic potential. More importantly, they should also allow improvements in the design of currently used retroviral vectors for human gene therapy as well as other gene-based approaches to treatment so as to minimize the chances of recombination, which is currently considered the most significant setback in using retroviral vectors in clinical trials.

## Figures and Tables

**Table 1:** Summary of the packaging determinants mapped to the R/U5, 5’ untranslated region (5’ UTR)* and *gag* gene of different retroviruses.

Virus	Length of 5’ UTR*	5’ UTR* and R/U5 Sequences Required	*gag* Sequences Required	Reference
HIV-1	~152 nts	Entire UTR, SL1 and SL3	<455 nts	[18,29]
HIV-2	~246 nts	140 nts	366 nts	[40,41,42,43]
SIVmac239	~231 nts	117–175 nts upstream of *gag* ATG (SL1, SL3 and SL4)	?	[29,44,45]
FIV	~270 nts	First 150 nts	100 nts	[46]
MPMV	~128 nts	First 50 nts + last 23 nts	100 nts	[47,48]
MMTV	~160 nts	Entire UTR	120 nts	[49]
BLV	~94 nts	Entire UTR	First 70 nts of *gag* (SL1 and SL2) + 132 nts (nt 1015–1147) in CA	[50,51,52]
HTLV-1	~94 nts	?	~70 nts in *gag* (SL1 and SL2)	[53]
MLV	~476 nts	350 nts (nt 215–565) of 5’ UTR	328 nts	[54,55,56]
SNV (REV)	~401 nts	180 nts (SL1 and SL2)	?	[57,58]
RSV	~278 nts	82 nts (O3 stem)	?	[59,60,61]
HFV	~100 nts	Entire UTR	?	[34,35,36]

5’ UTR: 5’untranslated region here is defined as the region between the start of the primer binding site and before *gag* ATG. HIV: human immunodeficiency virus; SIV: simian immunodeficiency virus; FIV: feline immunodeficiency virus; MPMV: Mason-Pfizer monkey virus; MMTV: mouse mammary tumor virus; BLV: bovine leukemia virus; HTLV-1: human T-lymphotropic virus type 1; MLV: murine leukemia virus; SNV: avian spleen necrosis virus; REV: Reticuloendothelial virus; RSV: Rous sarcoma virus; HFV: human foamy viruses; SL: stem loop; CA: capsid.

**Table 2:** Summary of RNA cross-/co-packaging and propagation among retroviruses.

Protein	HIV-1		HIV-2		SIV		FIV		MPMV		MMTV		SNV/REV		MLV		RSV		BLV	
RNA	PACK	PROP	PACK	PROP	PACK	PROP	PACK	PROP	PACK	PROP	PACK	PROP	PACK	PROP	PACK	PROP	PACK	PROP	PACK	PROP
**HIV-1**			[40,137]	[40,137]	ND	[30]	[161]	[161]	[24]	[24]	[125]*	ND	[28]	[28]	[110]	[30]				
**HIV-2**	[40,137]	[40,137]			[137]	[137]														
**SIV**	[29,137]	[29,137]	[137]	[137]			[161]	[161]	[24]	[24]										
**FIV**	[161]	[161]			[161]	[161]			[24]	[24]										
**MPMV**	[24]	[24]			[24]	[24]	[161]	[161]			[25]	[25]								
**MMTV**	[125]	ND							[25]	[25]					[163]	[163]				
**SNV**	[28]	[28]													[138]	[138]				
**MLV**	[110]*	ND			ND	[30]							[31,58], [121]*, [138,141]	[31,58], [121]*, [138,141]			[119]*	[119]*	[52]	ND
**RSV**																				
**BLV**																				

Green: Yes; Red: No; *: RNA packaging tested in a chimeric Gag context; Grey: RNA packaging and propagation using proteins from the same virus; ND: not done.
